# Middle ear biofilm and sudden deafness - a light and transmission electron microscopy study

**DOI:** 10.3389/fneur.2024.1495893

**Published:** 2024-12-13

**Authors:** Hao Li, Karin Staxäng, Hanif M. Ladak, Sumit Agrawal, Helge Rask-Andersen

**Affiliations:** ^1^Department of Surgical Sciences, Otorhinolaryngology and Head and Neck Surgery, Uppsala University, Uppsala, Sweden; ^2^The Rudbeck TEM Laboratory, BioVis Platform, Uppsala University, Uppsala, Sweden; ^3^Department of Otolaryngology-Head and Neck Surgery, Western University, London, ON, Canada; ^4^Department of Medical Biophysics, Western University, London, ON, Canada; ^5^Department of Electrical and Computer Engineering, Western University, London, ON, Canada

**Keywords:** human, round window, biofilm, occult infection, synchrotron phase-contrast imaging, sudden deafness

## Abstract

**Background:**

There still exists controversy about whether the healthy human middle ear mucosa is sterile or if it may harbor a diverse microbiome. Considering the delicacy of the human round window membrane (RWM), different mechanisms may exist for avoiding inner ear pathogen invasion causing sensorineural deafness. We re-analyzed archival human RWMs using light and transmission electron microscopy after decalcification to determine if bacteria are present in clinically normal human middle ears. We also searched for the presence of inborn immune defensive mechanisms within the round window niche (RWN), as previously reported in non-human primate ears.

**Materials and methods:**

Five round window niches, removed and directly fixed at transcochlear petroclival meningioma surgery, were re-investigated after ethical permission using light and transmission electron microscopy. The morphology of the RWM, including its bony attachment and pseudomembrane outline, was analyzed. Moreover, 64 human temporal bones were investigated using synchrotron phase-contrast imaging (SR-PCI) aiming to identify potentially “hidden” spaces, including the RWN potentially harboring infectious material.

**Results:**

Histologic evidence of free-living bacteria and biofilm was found in 40% of RWNs in seemingly “healthy” middle ears. The RWM in these ears was pathologically changed with repealed epithelial and intercellular junctional integrity. Putative membranous defense machinery consisted of a lymphatic drainage system together with free phagocytic cells seemingly serving to protect the inner ear from alleged pathogens. Synchrotron analyses showed that a pseudomembrane was present in the human round window niche (RWN) in 80% of the specimens, of which 20% were complete. In 3%, the RWN contained dense tissue or serous fluid plugs partly obstructing the RWN. Infralabyrinthic clefts and tympanomeningeal fissures (Hyrtl’s fissure) were occasionally enclosed by delicate membranes near the round window. These may represent predilection sites for “hidden” infections potentially endangering inner ear function, particularly in connection with round window surgery.

**Conclusion:**

Considering the fragility of the normal human RWM, we speculate that occult colonies of biofilm may be a factor in surgeries involving the RWM, sensorineural hearing loss, and hearing preservation/fibrosis following cochlear implantation, and more controversially in hidden perilymph leaks causing sudden deafness and labyrinthine pathology.

## Introduction

Due to its proximity to infection-prone areas, the human cochlea and inner ear are potentially challenged by invading pathogens via the Eustachian tube. Moreover, the round window membrane (RWM) is thin and fragile serving important auditory functions. The RWM is protected and hidden behind a bony overhang and invariably by an extra-, or pseudomembrane that may serve as an extra shield.

In a recent study using synchrotron phase-contrast imaging (SR-PCI) and 3D rendering, we found that the round window niche (RWN) is entirely closed against the middle ear with isolated spaces in 20% of an unselected collection of human temporal bones (submitted O&N 2024). We speculate whether such hidden spaces could potentially house occult pathogens endangering inner ear function, particularly in connection with RWM surgery.

In this study, we re-analyzed archival well-preserved human cochlear tissue using light (LM) and electron microscopy (TEM) (1, 2). It consisted of RWMs where the middle ears were considered “healthy” with normal eardrums investigated before transotic removal of petroclival meningioma. Bacteria and biofilms were discovered in the RWNs in two out of five specimens together with signs of an inherent biological defense response. Normally, the RWM was found to be 12–20 microns thick with an epithelial cell layer consisting of shallow tight junction strands. The significance of these findings in connection with round window surgery and inner ear conditions is discussed.

## Materials and methods

### Light and transmission electron microscopy

Five archival human RWMs from cochlear specimens removed in patients operated on for petroclival meningioma using the transcochlear approach were re-analyzed. Preoperatively, all ear drums were considered normal with no signs of external or middle ear infections or other middle ear pathology notable by the surgeon. All five specimens were semi-thin sectioned and ultrathin-sectioned for transmission electron microscopy (TEM). Surgical procedures, fixation, and processing of the tissue for LM and TEM were earlier described (2). Semi-thin sections were stained with toluidine blue, followed by ultrathin sectioning of areas of interest. The sections were stained in lead citrate and uranyl acetate and examined at 80 kV with a Tecnai™ G2 Spirit TEM (Thermo Fisher/FEI Company, Eindhoven, NL). Images were captured with an ORIUS^™^ SC200 CCD camera (Gatan Inc., Pleasanton, CA, United States) using Gatan Digital Micrograph software.

### Synchrotron phase-contrast imaging

To search for hidden middle ear spaces, potentially housing occult bacterial infections, 64 cadaveric human temporal bones with no known otologic disease underwent SR-PCI at the Canadian Light Source Inc. (Saskatoon, SK, Canada) using the Biomedical Imaging and Therapy beamline (05ID-2). The gender and age of the donors were unknown. A total of 13 pairs of bones with left and right ears were used. The SR-PCI technique used was previously described (3–5). Open-source medical imaging software, 3D Slicer (www.slicer.org, version 5.0.3; (6)), was used to segment and create 3D representations of the human specimens for analyses of the human RW and its niche. A more systematic presentation of the SR-PCI results is shown in a separate study (O&N, submitted September 2024).

## Results

**Figure 1:**
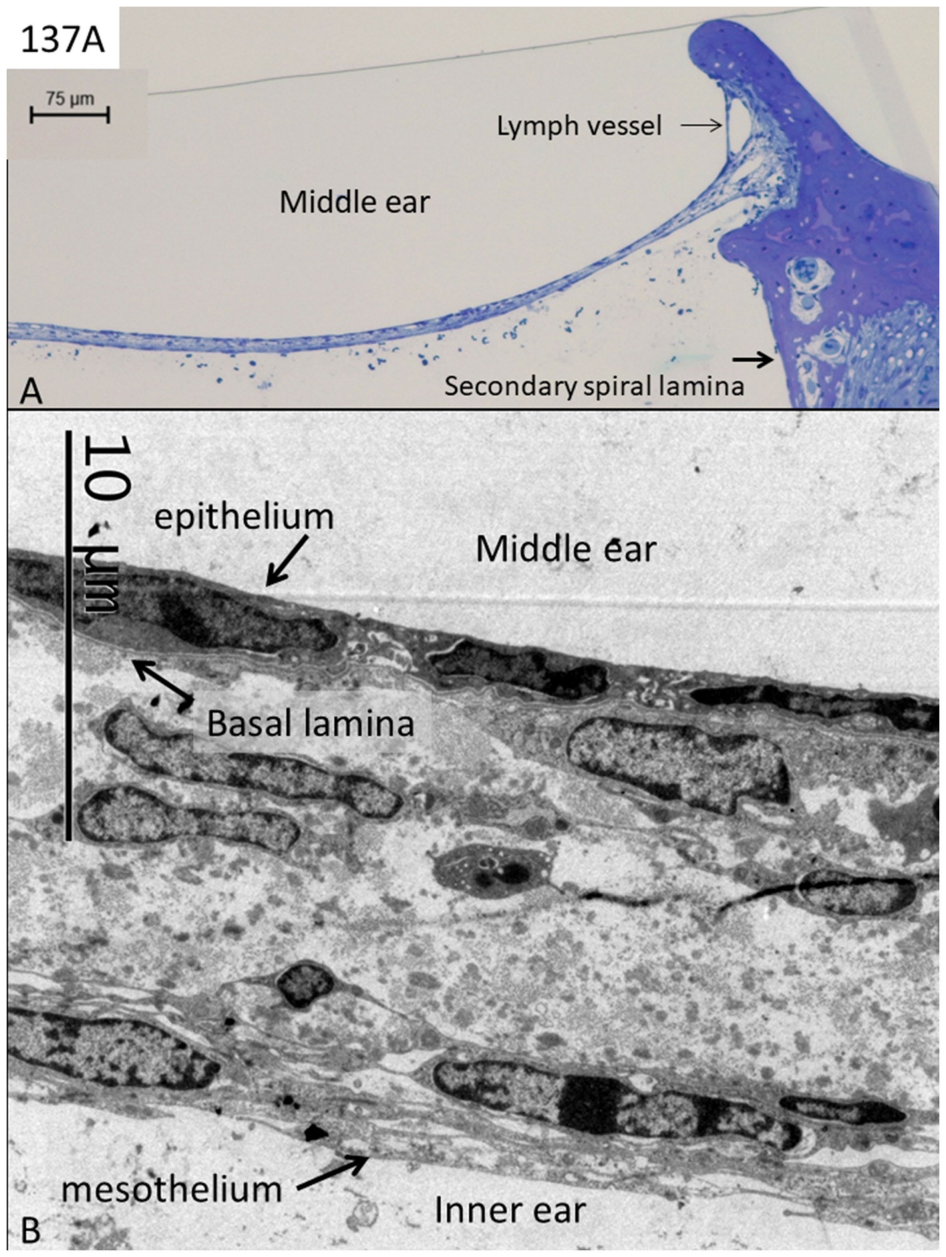
Light **(A)** and electron microscopy **(B)** of a normal human round window. The thickness of the membrane is approximately 12–20 μm, while the epithelial layer can be as thin as 50 nanometers. Its inner surface consists of a mesothelium layer lacking a basal lamina. The middle stroma contains cells producing collagen and elastic fibers explaining its great elasticity.

**Figure 2:**
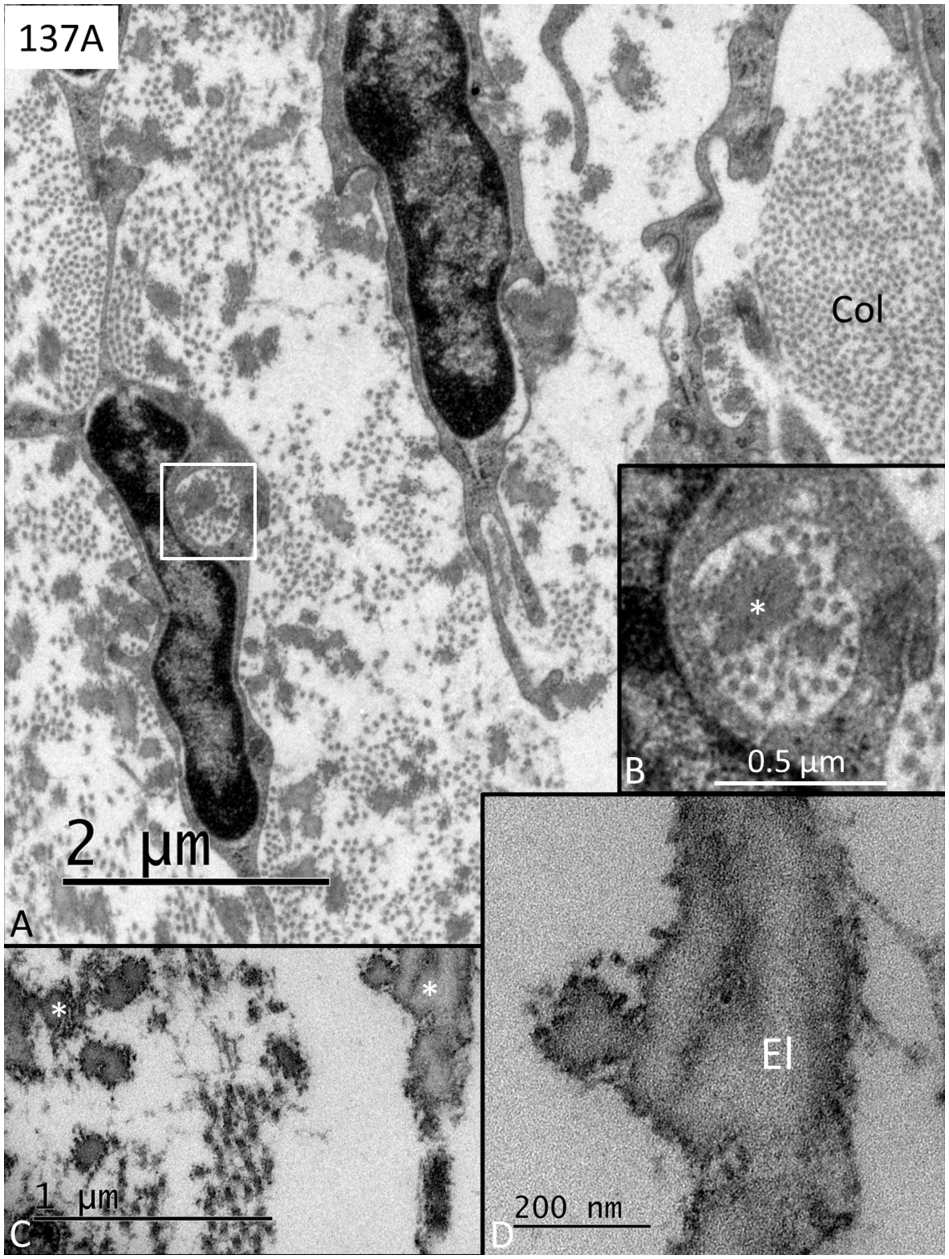
**(A)** Transmission electron microscopy of a human RWM shows fibrocytes producing collagen (col) and elastic fibers. The framed area in **A** is shown in higher magnification in **B** with enclosed secretory products of both elastic fibers (*) and collagen fibers. **(C)** Elastic fibers of different sizes (*). **(D)** Higher magnification shows the fine structure of the elastic fibers (El).

**Figure 3:**
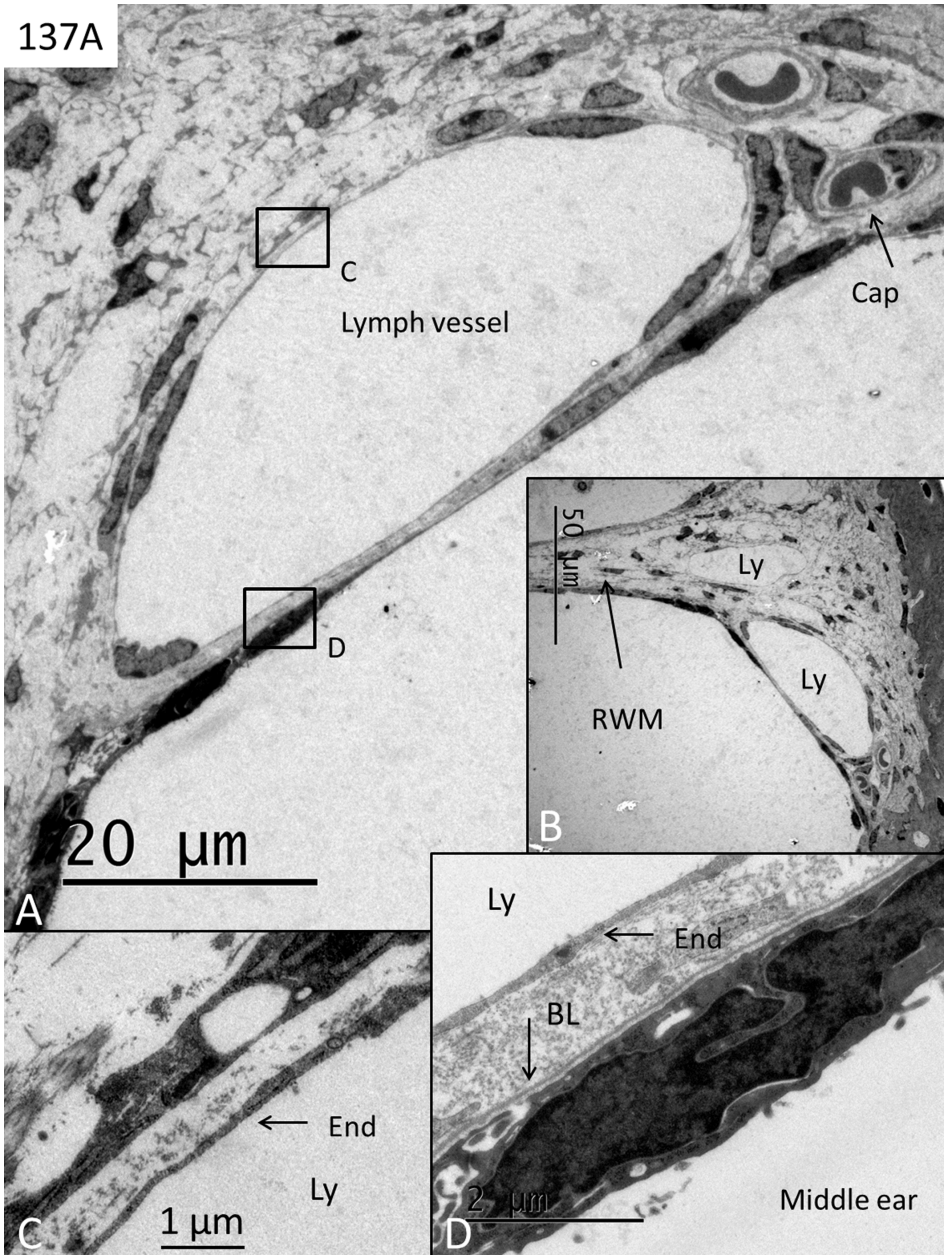
**(A,B)** TEM of the RWM at the lateral bony insertion with several thin-walled lymph vessels (Ly) surrounded by capillaries (cap). **(C)** Framed area in **A** is shown in higher magnification. The lymph vessel endothelium (end) is thin with no basal lamina. **(D)** Framed area in **A** shows the thin endothelium (End) of the lymph vessel facing electron-dense epithelial cells of the middle ear mucosa. BL; basal lamina. End; endothelium of the lymph vessel.

**Figure 4:**
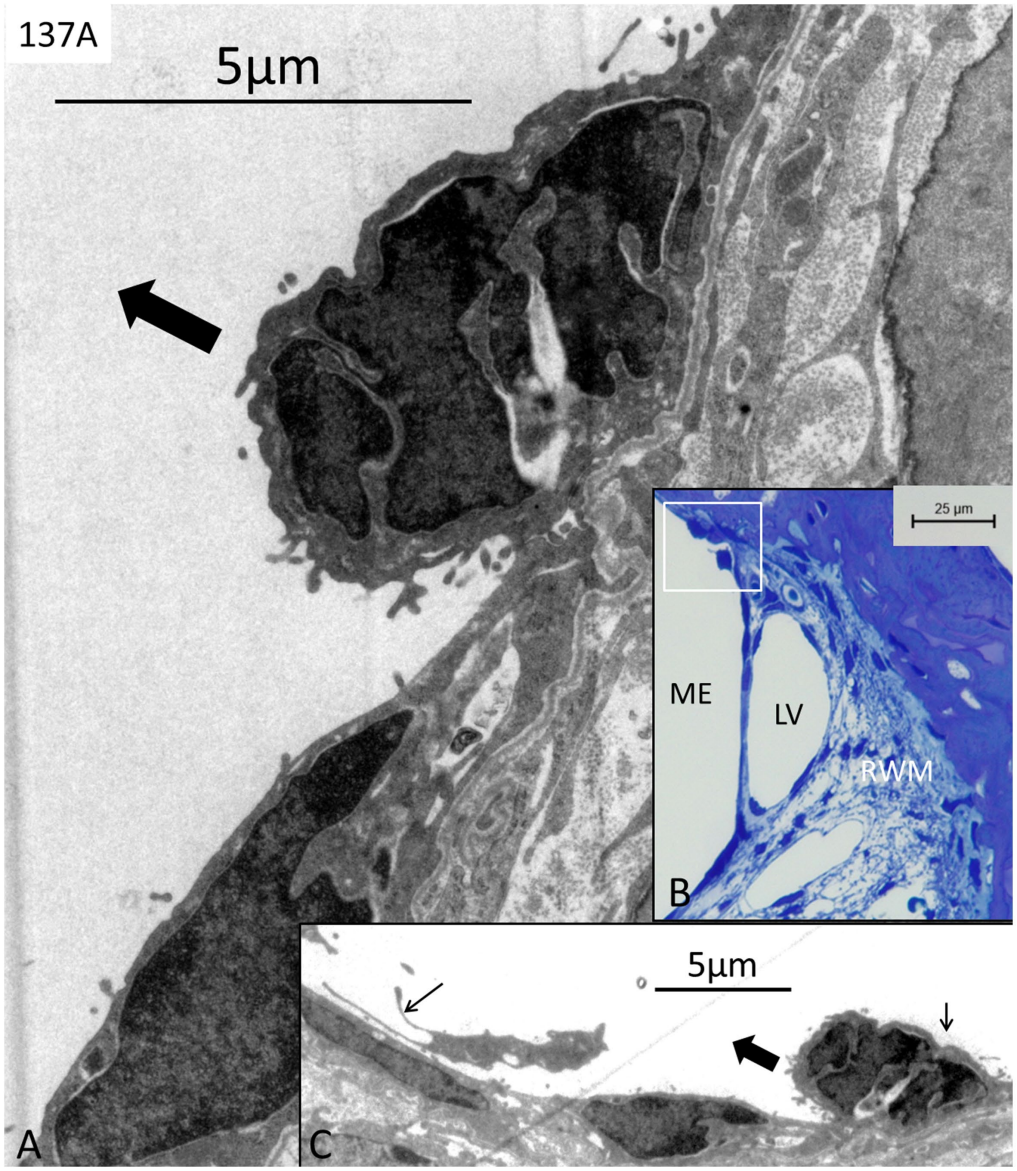
**(A)** TEM of dark middle ear epithelial cells of the round window near the bony wall in a bacteria-free specimen (137A) (framed area in **B**). **(C)** Adjacent thin section shows the same cell seemingly loosening from the epithelium (bold arrow). The cell conceivably undergoes cell division (small right arrow in **C**). A free dendritic cell can also be seen in the middle ear (small left arrow). LV; lymph vessel. RWM; round window membrane. ME; middle ear.

Three RWM specimens (134A, 136A, and 137A) displayed no signs of any bacteria or biofilm, and cell structures were excellently preserved (Figures 1–4). The RWM thickness was approximately 12 μm at the thinnest point and widened triangular at the RWM annulus at the bony attachment (Figure 1A). The RWM epithelium facing the middle ear was flat with shallow intercellular tight junctions while the mesothelium facing scala tympani lacked intercellular junctions as well as a basal lamina (Figure 1B). The stromal tissue contained mesenchymal cells showing active signs of collagen and elastic fiber production and secretion (Figure 2). The RWM connective tissue contained fibrocytes, a few free cells, and lymphatic vessels that enlarged at the annulus surrounded by several blood capillaries (Figures 3A,B). Lymphatics were thin-walled, had no gaps, and lacked a typical basal lamina (Figures 3C,D). The epithelial cells facing the middle ear varied greatly in thickness, and at the annulus, these cells increased in size, were more electron-dense, and occasionally gave the impression of transforming into free cells (Figures 4A–C). Free dendritic-like cells conceivably representing macrophages could also be noted (Figure 4C).

**Figure 5:**
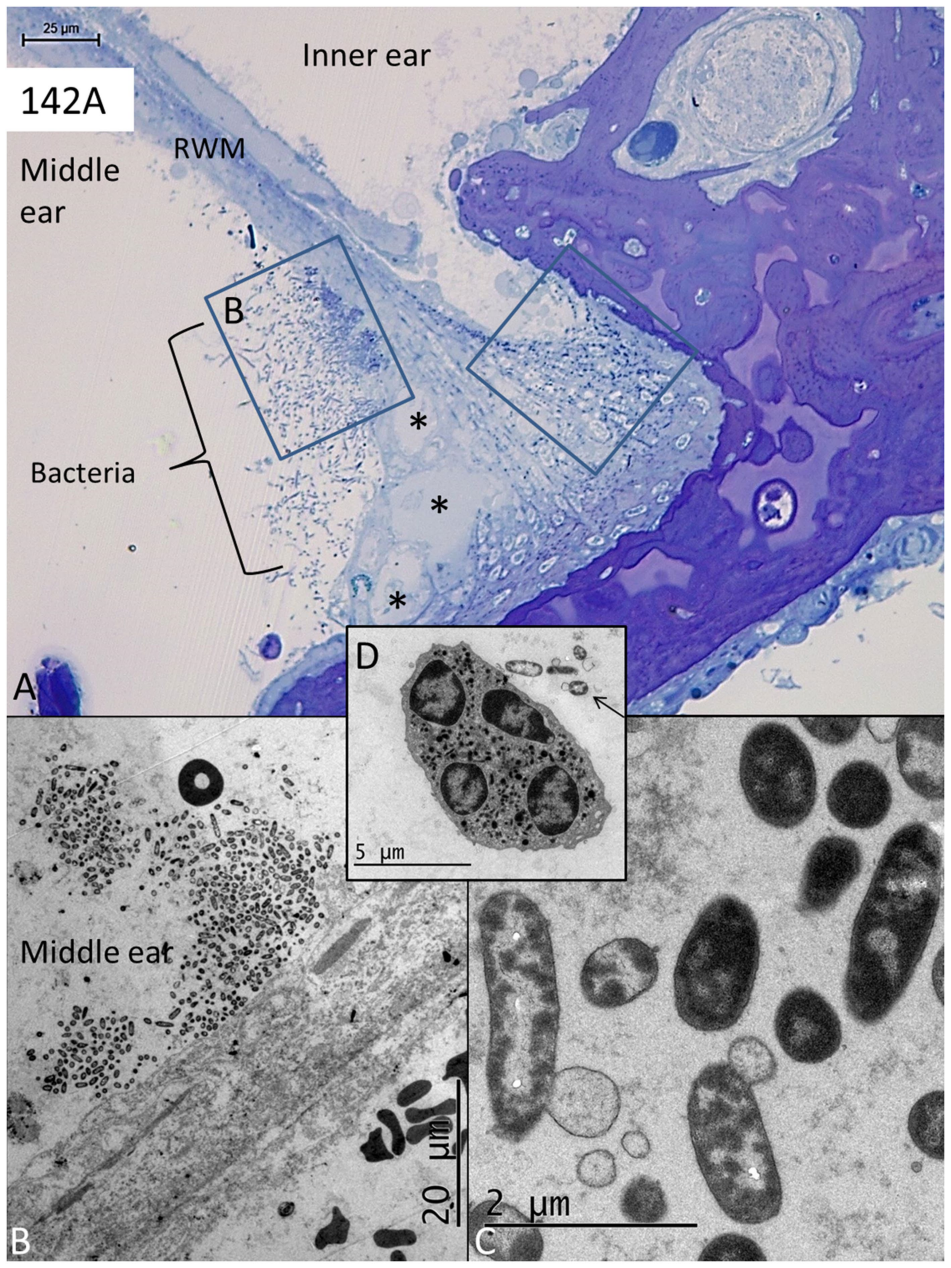
**(A)** Light microscopy of a human RWM at the lateral bony attachment. Bacterial pathogens (coccobacilli) are present in the middle ear facing the RWM and in the tissue stroma near the bony insertion (framed areas). Bacteria in the left frame are magnified in **B**. There are several dilated lymph vessels in the RWM (asterisks). **(C)** Bacteria are shown in higher magnification. **(D)** A neutrophil is engaged in bacteria phagocytosis (arrow).

**Figure 6:**
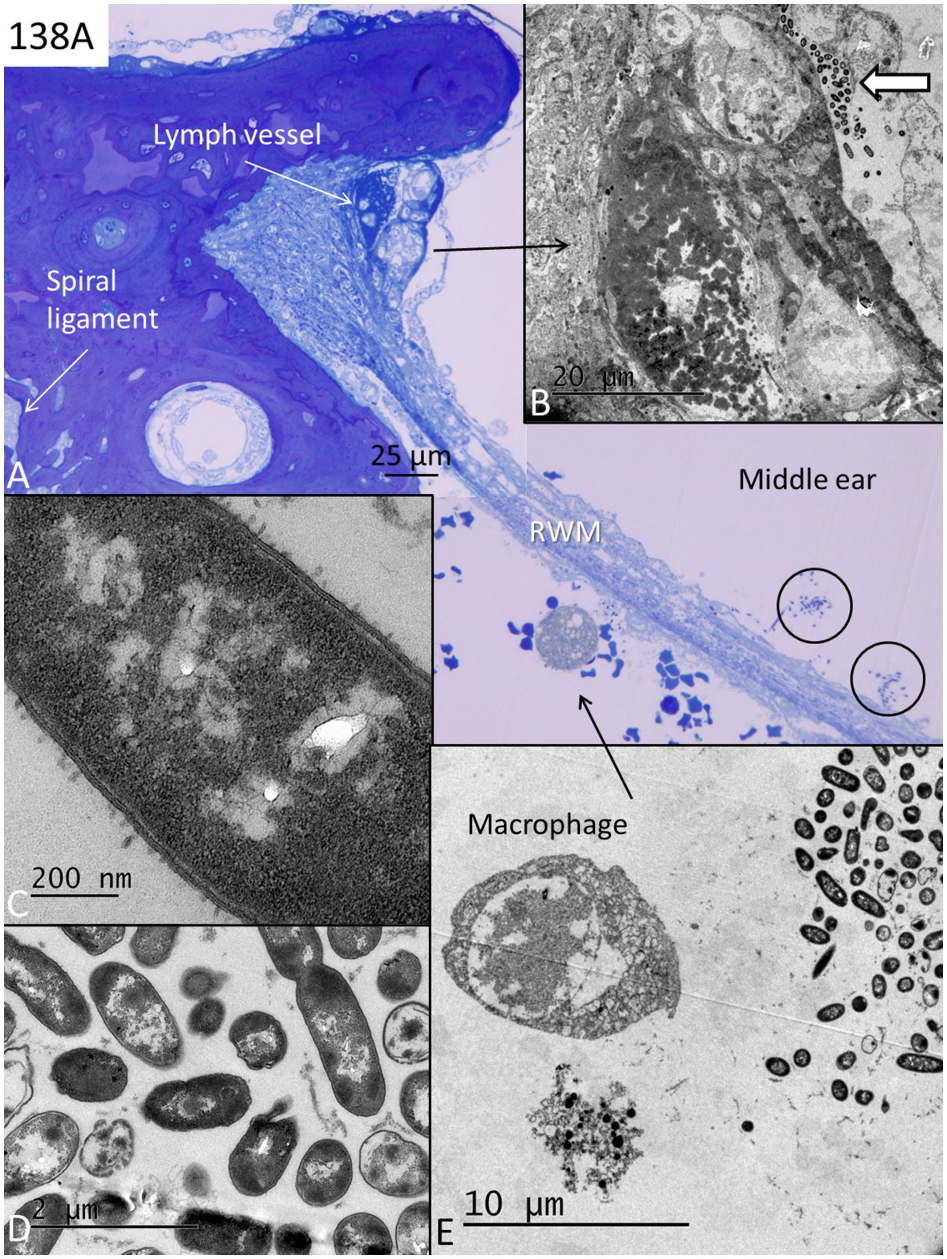
**(A)** Light microscopy of the lateral bony insertion of the RWM in another specimen with pathogens on both sides of the edematous membrane (encircled in the middle ear). A macrophage appears on the scala tympani side engaged in bacteria phagocytosis (toluidine and osmium staining). **(B)** TEM of the lymphatics shown in **A** enrolled in bacteria phagocytosis, entrapment (white arrow), and transportation in the lymph vessel containing secretory masses. **(C)** A coccobacilli seen in higher magnification. The bacteria cell wall has a pentalaminar structure. **(D)** Coccobacilli with one diplobacillus at upper right. **(E)** TEM of the area shown in **A** (arrow). One degenerated cell contains degraded bacteria. RWM; round window membrane.

In two specimens (138A and 142A), there were free and conglomerates of bacteria and biofilm facing the external surface of the RWM, as well as bacteria within the membrane (Figures 5A–D, 6A–E). The bacteria were ultrastructural identical in both ears. They represented coccobacilli having a diameter of approximately 0.5–1 μm. Some bacteria penetrated deep into the annulus region (Figures 5A,C). There was a leucocyte response with macrophages and a few neutrophils and signs of active phagocytosis with macrophages containing degraded bacteria (Figures 5B,D). The lumen of the lymphatics was filled with electron-dense secretory material, a few leucocytes, and degraded bacteria (Figures 6A,B). The bacterial wall had a typical pentalaminar structure (Figure 6C). Diplobacilli could also be seen (Figure 6D). A few bacteria had entered into the scala tympani, and the RWM was edematous partly undergoing degeneration (Figure 6E). The organ of Corti and the lateral wall tissue at the RWM were also affected by the infection, showing signs of cell degeneration. However, whether cell structure higher up in the cochlea was affected could not be evaluated in these specimens.

**Figure 7:**
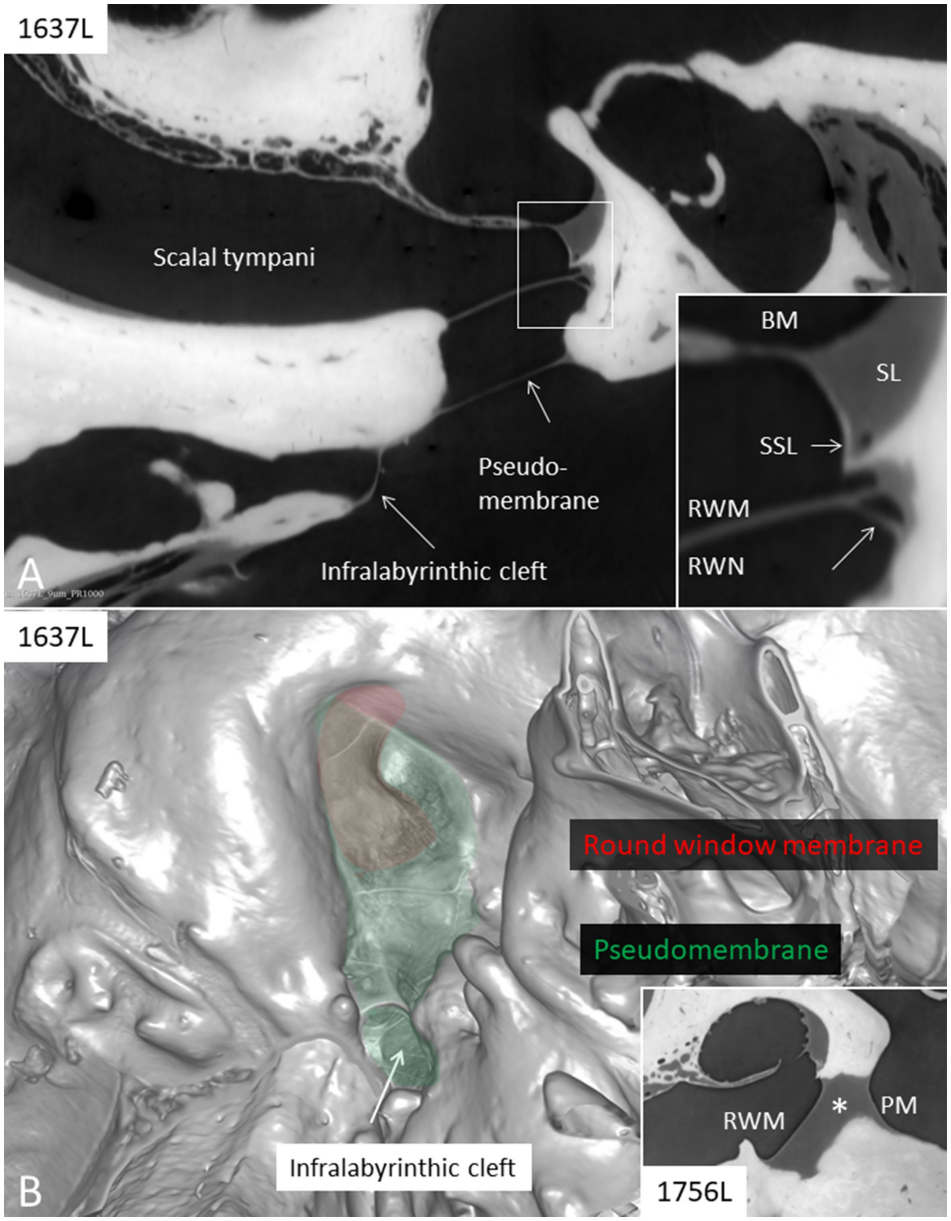
**(A)** SR-PCI (1637L) of a left human ear with pseudomembrane (PM) closing the round window niche that is completely aerated. At the hypotympanum there is an infra-labyrinthine cleft also closed by a membrane. Framed area is shown in higher magnification in the inset. A vessel is shown at the angle between the round window membrane (RWM) and the wall of the round window niche (RWN, lower arrow). **(B)** 3D rendering (1637L) shows the pseudomembrane and the round window membrane as well as the entrance of the infra-labyrinthine cleft. Inset shows, in a different specimen (1756 L), a serous fluid plug (*) between the RWM and pseudomembrane. BM; basilar membrane. SSL; secondary spiral lamina. SL; spiral ligament.

The synchrotron radiation phase-contrast imaging (SR-PCI) of human temporal bones and the 3D reconstructions of the round window region were accomplished in 66 human cadaveric temporal bones. The complete results are published in a separate paper (submitted for publication in O&N, 2024). Synchrotron analyses showed that a pseudomembrane was present in the RWN in 80% of the specimens, of which 20% were completely closed from the middle ear. The SR-PCI of a left human ear with pseudomembrane closing the RWN is shown in Figures 7A,B. It also demonstrates an infralabyrinthic cleft closed by a thin membrane. A 3D rendering shows the pseudomembrane as well as the entrance of a widened space beneath the cochlea. In 3%, the RWN contained dense tissue or serous fluid plugs partly obstructing the niche. A serous fluid plug can be seen between the round membrane and pseudomembrane in the RWN (Figure 7B, inset).

## Discussion

There is still some controversy over whether healthy human middle ear mucosa is sterile or may harbor a diverse microbiome. The results may depend on the techniques and sensitivity of the methods used (7–11). A molecular study revealed no evidence of substantial bacterial populations in the healthy middle ear (7). Conversely, another similar study of surgical samples collected from normal middle ear mucosa provided ample evidence of bacterial populations, with the most abundant being Proteobacteria, Actinobacteria, Firmicutes, and Bacteroidetes, which differed between children and adults (8). A majority of mucosal specimens collected from the promontory at cochlear implantations (CIs) displayed inflammatory and dispersed bacteria with fragments of biofilms (10). However, another similar study showed no biofilms in specimens obtained from patients undergoing CI surgery (12).

Synchrotron phase-contrast imaging and 3D rendering present novel sophisticated information on the human temporal bone anatomy (3). The human middle ear consists of complex, partially separated outpouchings where stagnated secretions and contagious material could potentially be concealed. Spaces such as the RWN, tympanomeningeal fissure [Hyrtl’s fissure, Mudry (13)], and infralabyrinthic clefts near the round window could be colonized by occult microorganisms. Conceivably, this could explain the different results obtained from cultivating middle ear tissue. These regions may be potential risks during inner ear surgery, particularly during RWM surgery, which critically challenges the surgically opened inner ear. Even though, the present results cannot substantiate that the closed spaces in the middle ear house hidden infectious material, it may highlight the importance of irrigating and cleaning the RWN before perforating the RWM to avoid contagions being transmitted into the inner ear during surgery.

The present study showed that bacteria biofilms may be present in the RWN in non-diseased ears. This may be relevant to consider, not only for avoiding pathogen invasion during round window surgery but also for their potential role in causing perilymph leak and sensorineural deafness. In the affected specimens, the RWM was edematous and the epithelial barrier was partly down-broken, which could result in noxious substances reaching perilymph. Inflammatory changes of the RWM could lead to a breach of the membrane barrier and leak of perilymph resulting in sudden and hidden sensorineural deafness. The cause of sudden deafness is still much of an enigma and is under debate. Hidden perilymph leaks have recently been debated as a possible cause where fistula repair is feasible (14). Novel diagnostic tests using biomarkers and high-resolution imaging may assist in improved diagnosis of these difficult and rare conditions. Vascular and immune-mediated causes are suspected and anti-inflammatory drugs such as corticosteroids are used, and even injected intra-tympanic.

**Figure 8:**
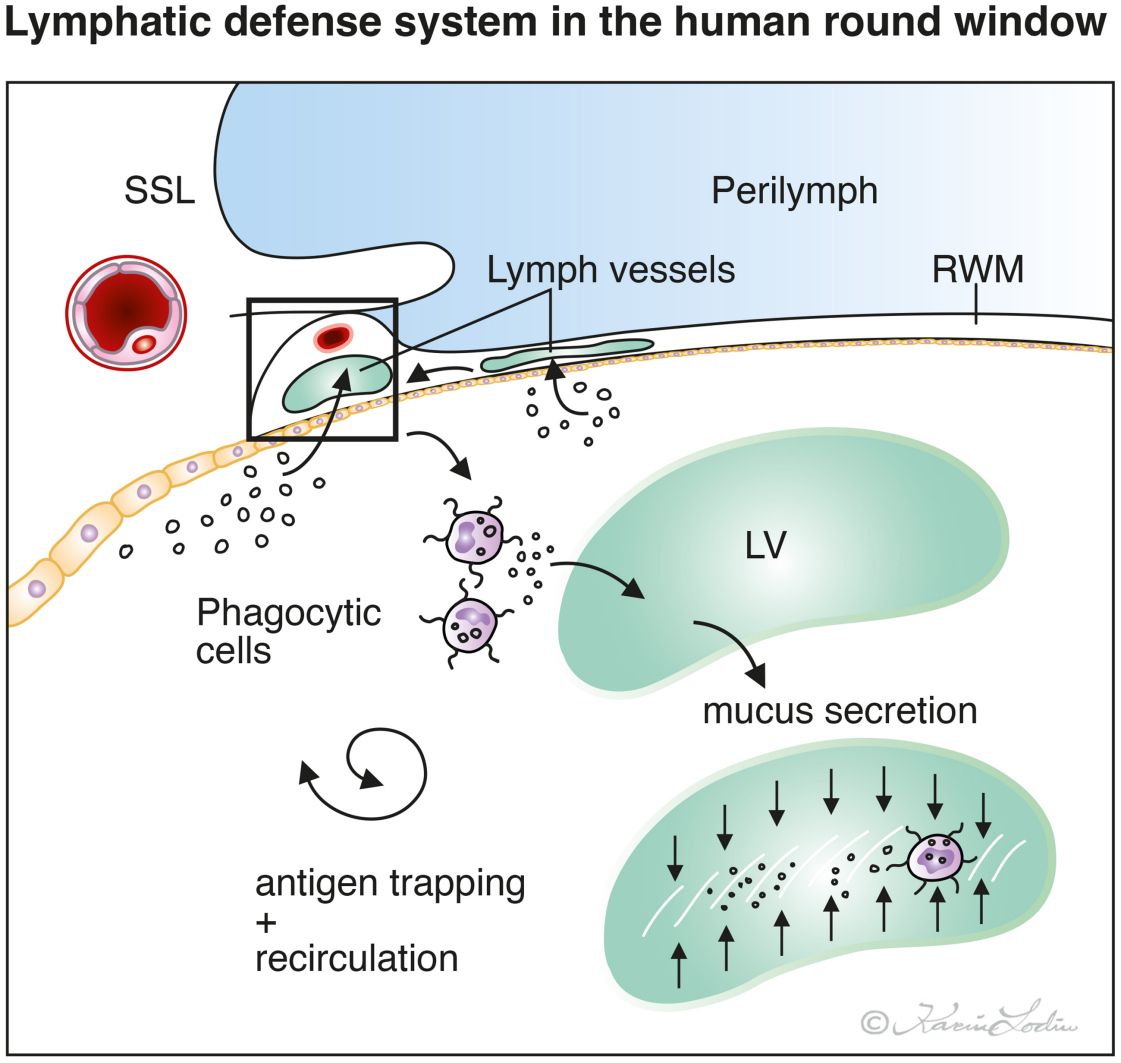
Principle drawing of feasible lymphatic defense system present in the human round window membrane (RWM) and niche (RWN). Microorganisms and foreign substances reaching the middle ear may be trapped by mucus secretion and immune cells, processed and recirculated along the lymphatics and blood vessels within the RWM. Thereby, the RWM may act to protect the inner ear from entering pathogens. SSL; secondary spiral lamina. LV; lymph vessel.

Our results show that the human RWM may be endowed with a lymphatic drainage system involved in the protection of the inner ear. The RWM is well vascularized and contains thin-walled lymph vessels identified by their endothelial cell characteristics and absent basal lamina. They can be perceived in the thin RWM partitions and at the membrane ring (annulus). Similar findings were made in the cynomolgus monkey and described in an earlier publication (15). Sophisticated immune-cellular protection of the inner ear from invading pathogens appears to be present in the RWN. In the cynomolgus monkey, strategically located glands were localized at the peripheral rim of the RWM surrounded by blood and lymph vessels. Hence similarly, foreign material may be trapped in the RWN by the secretion of glycoproteins recirculated and disposed across lymphatics in humans. Immunoglobulins and chemotactic attraction of leucocytes and macrophages may follow to clear this critical space to circumvent the entering of noxious material into the vulnerable inner ear (Figure 8). Our findings could suggest that vigilant attentiveness may be motivated to scrutinize hidden RWM ruptures in patients with idiopathic sudden sensorineural hearing loss in the future.

### Limitations of this study

Tissue histologically analyzed was removed in patients with petroclival meningioma, a condition that could influence inner ear homeostasis and immune conditions. The ear drum and middle ears were considered “healthy” when investigated before transotic removal. Although there were no signs of tumor invasion, all patients showed pathologic changes in the vicinity of the inner ear that could have influenced the cell anatomy. Repeated morphological analyses, including electron microscopy of the human cochlea, had not previously revealed bacteria infiltration.

## Data Availability

The original contributions presented in the study are included in the article/supplementary material, further inquiries can be directed to the corresponding author.
